# Psychological wellbeing and the association with burnout in a cohort of healthcare workers during the COVID-19 pandemic

**DOI:** 10.3389/frhs.2022.994474

**Published:** 2022-10-25

**Authors:** Jacqueline Bannon, Charlesnika T. Evans, Melanie Freedman, Cerina Lee, Thanh Huyen Vu, Amisha Wallia, John T. Wilkins, Judith T. Moskowitz, Lisa R. Hirschhorn

**Affiliations:** ^1^Department of General Medicine, Northwestern Memorial Hospital, Chicago, IL, United States; ^2^Center for Health Services and Outcomes Research, Feinberg School of Medicine, Institute for Public Health and Medicine, Northwestern University, Chicago, IL, United States; ^3^Department of Preventive Medicine, Feinberg School of Medicine, Northwestern University, Chicago, IL, United States; ^4^Department of Veterans Affairs, Center of Innovation for Complex Chronic Healthcare, Edward Hines, Jr. VA Hospital, Hines, IL, United States; ^5^Department of Medical Social Sciences, Feinberg School of Medicine, Northwestern University, Chicago, IL, United States; ^6^Institute for Public Health and Medicine, Center for Education in Health Sciences, Northwestern University, Chicago, IL, United States; ^7^Department of Medicine, Division of Endocrinology, Metabolism, and Molecular Medicine, Feinberg School of Medicine, Northwestern University, Chicago, IL, United States; ^8^Department of Medicine, Division of Cardiology, Feinberg School of Medicine, Northwestern University, Chicago, IL, United States

**Keywords:** healthcare worker (HCW), burnout—professional, positive psychology, depression, anxiety, cohort study

## Abstract

Burnout, depression, and anxiety are prevalent among healthcare workers (HCWs) during the COVID-19 pandemic and have been previously shown to contribute to poor health outcomes and reduced quality of care. Positive psychological constructs such as positive affect and meaning and purpose are related to resilience in the face of significant stress. No studies have examined these associations among a cohort of HCWs during this pandemic. The purpose of this study was to examine the association of depression, anxiety, positive affect, and meaning and purpose with burnout among HCWs during the COVID-19 pandemic. We utilized data from a cross-sectional survey conducted between September 29-December 8, 2021, among a cohort of 2,411 HCWs from a large, tertiary academic health care system in the Chicago area. We employed the Patient-Reported Outcomes Measurement Information System (PROMIS) measures for depression, anxiety, positive affect, and meaning and purpose and burnout was measured by the Oldenburg Burnout Inventory (OLBI). The majority (80.88%) of HCWs in this study identified as White, Non-Hispanic race/ethnicity, female sex (82.37%), and roughly one third were between ages 30–39 years old (30.98%). Registered nurses (26.96%) accounted for the largest single occupation group. The mean burnout score was 36.87 (SD = 7.65), with 53.38% of participants classified as having burnout, and registered nurses demonstrating the highest proportions of burnout (63.54%). Higher depression (coef = 0.15, SE = 0.03, *p* < 0.001) and anxiety (coef = 0.25, SE = 0.02, *p* < 0.001) scores were associated with higher burnout in multivariable linear regression models. Increased positive affect (coef= −0.19, SE= 0.02, *p* < 0.001) and meaning and purpose (coef= −0.12, SE= 0.01, *p* < 0.001) scores were significantly associated with reduced burnout. Positive affect and meaning and purpose were inversely associated with burnout among a cohort of HCWs during the COVID-19 pandemic. Previous studies of positive affect and meaning and purpose suggest the potential buffering effect that these indices may have on burnout. Future research is needed to examine the effect of positive affect and meaning and purpose on mitigating the negative impacts of burnout, depression, and anxiety among HCWs as they cope with the stress of the COVID-19 pandemic and beyond.

## Introduction

Burnout is an ongoing, significant threat to healthcare worker (HCW) well-being and to health systems' abilities to deliver quality, people-centered care (1–4). Burnout is characterized by emotional exhaustion (feeling emotionally drained by contact with patients), depersonalization (extreme detachment from patients), and lack of personal accomplishment (feelings of incompetence and lack of job success) (5–7). Burnout has been linked to poor health outcomes for HCWs- including depression, cardiometabolic risk behaviors (e.g. reduced physical activity, cigarette smoking), increased alcohol consumption, and sleep disruptions (8–11)—and the work environment and patients they care for, such as through increased medical errors, absenteeism, and poor quality of care (12–15).

Studies of burnout have highlighted the severe mental health impacts to HCWs amid the COVID-19 pandemic (16–18). Several literature reviews and meta-analyses have noted increased prevalence of depression, anxiety, sleep disturbances, post-traumatic stress, and other psychological disorders among HCWs since the start of the pandemic (19–24). However, studies have less frequently examined domains of positive psychological wellbeing and their associations with HCW burnout. Additionally, there are few evidence-based interventions to reduce burnout, anxiety, and depression among HCWs. Of available evidence-based interventions for HCWs, these interventions largely focus on mindfulness or meditation-based interventions rather than broader psychological domains of wellbeing (25–28).

Growing evidence from groups experiencing a variety of types of significant life stress, suggests that the ability to maintain positive psychological wellbeing in the context of stress may be protective against mental and physical health consequences of stress and burnout (29–32). Psychological wellbeing has traditionally been conceptualized to include both hedonic, or feelings of positive affect, and eudaimonic, or a sense meaning and purpose in life, domains (33–35). Among HCWs, positive affect has been associated with increased creative problem solving and job satisfaction, and these aspects of psychological wellbeing may help to buffer the stress of the COVID-19 pandemic and reduce the likelihood of burnout (36–38). To that end, a focus on wellbeing has recently been prioritized by the U.S. Surgeon General, as health systems must promote meaning and purpose in work to reduce burnout as HCWs provide life-saving care (39).

In the present study, we examine the associations of depression, anxiety, positive affect, and meaning and purpose with burnout among a cohort of HCWs in the US during the COVID-19 pandemic. We hypothesize that burnout will be positively associated with depression and anxiety, and inversely associated with positive affect and meaning and purpose. We will also examine the extent to which demographic factors (e.g. age, biological sex), occupational role, degree of patient contact, and occupational COVID-19 exposure, provide insights into the association between psychological health and burnout.

## Methods

### Study design and participants

This was an analysis of data from the Northwestern Medicine Healthcare Worker SARS-CoV-2 Serology Study, a prospective cohort study of healthcare workers from a large, tertiary academic health care system. The study is described in more detail elsewhere but briefly, includes HCWs from 10 hospitals, 18 immediate care centers, and 325 outpatient practices in the Chicago area and surrounding Illinois suburbs (40–42). From May 28 to June 30, 2020, 38,127 Northwestern Medicine HCWs were invited in to participate in an employer-sponsored benefit that provided free SARS-CoV-2 serological testing, for which all HCWs were eligible. A total of 18,985 (49.8%) participated in the employer-sponsored serological benefit. HCWs enrolled in the benefit included 79.6% women and 74.9% non-Hispanic White, 9.7% Asian, 7.3% Hispanic, and 3.1% non-Hispanic Black workers, with a mean age was of 40.6 (SD:12.0) years old, and nurses represented the largest occupational group (*n* = 1794) (41). Those that attended the serologic testing were recruited to enroll in the Northwestern Medicine Healthcare Worker SARS-CoV-2 Serology Study, a 1-year cohort study, which included monthly, self-administered, online surveys related to COVID-19 exposures, testing, symptoms, and vaccine intentions. In the original 1-year cohort study, 6,510 HCWs enrolled in the study. In June 2021, cohort participants were recruited to continue in an extension cohort study in which 3,538 consented to enroll in the ongoing extension cohort. This present analysis was conducted on psychological health and burnout data collected from the participants who enrolled in the extension and completed a self-administered, online survey between September 29 and December 8, 2021.

### Measures

#### Descriptive variables

Participants were asked to report demographics (e.g. race/ethnicity, age, and sex), household size, and occupation (e.g. registered nurse, physician, phlebotomist, administration, etc.). We also asked about patient contact, exposure to procedures, place of work (including home) and self-reported COVID-19 infection. A total of 19 categorizations of occupational roles were included and can be viewed in Supplemental Table 1. For the main analyses, we collapsed occupations into four categories of registered nurse, physician, administrative roles, or other healthcare workers.

#### Burnout

Burnout was measured using the Oldenburg Burnout Inventory (OLBI), a validated 16–item inventory which measures two core dimensions of burnout: exhaustion and disengagement (from work) (43) and has strong validity and reliability among English-speaking workers in the United States (44). Responses to OLBI items range from 1 (totally disagree) to 4 (totally agree), and total scores range from 16 to 64. Consistent with work by Peterson et al. (45) we dichotomized burnout for descriptive analyses such that participants who had an average disengagement score of 2.1 or greater and an average exhaustion score of 2.25 or greater, were classified as having burnout. For our multivariable linear regression analysis, the OLBI score was kept continuous.

#### Psychological health

We utilized several measures from the Patient-Reported Outcomes Measurement Information System (PROMIS) computer adaptive tests (CAT) for measurement of psychological health, including anxiety, depression, positive affect, and meaning and purpose measures (46–49). All of the selected PROMIS psychological measures are examined as *T*-scores (*M* = 50, SD = 10) of the general population (50). We utilized cut points (e.g. high, average, low, etc.) to interpret the clinical significance of *T*-scores. For the PROMIS depression and anxiety measures, the cut points for normal limits of anxiety or depression are *T*-score values below 55, mild from 55 to 60, moderate from 60 to 70, and severe above 70. For PROMIS positive affect and meaning and purpose, T-score value cut points for very high scores greater than 70, high from 60 to 70, average from 40 to 60, low from 30 to 50, and very low with values less than 30. See Supplemental Figure 2 for interpretation of these measures. For all PROMIS measures, 3 *T*-score units can be interpreted as a meaningful difference in *T*-score values (51, 52).

#### Statistical analyses

We completed univariate analyses of all study data, including indices of central tendency (mean, median, 95% CI, etc.). *T*-tests for comparison of mean values of continuous demographic and health characteristics and chi-squared tests for comparisons of proportions for categorical characteristics were used to assess burnout as a dichotomous variable for descriptive analysis and to guide selection of covariates for our linear regression model. For the main analysis, OLBI scores were analyzed as a continuous outcome measure in a multivariable linear regression model to assess the association between depression, anxiety, positive affect, and meaning and purpose on burnout with selected covariates so that our analysis was sufficiently powered. Variables were included in the model based on the literature or significance at the *p* < 0.05 level in unadjusted analyses. The most parsimonious model was selected and reported. Moreover, respondents to the psychological health and burnout survey were compared to non-respondents among the 3,538 participants, to determine whether there were any significant differences in demographic characteristics and occupation. All analyses were conducted using Stata/IC (StataCorp) version 15 (53).

#### IRB

This study was approved by the Northwestern University Review board (STU00212515) prior to recruitment of HCWs, and all participants provided written informed consent at study enrollment.

## Results

A total of 2,619 of 3,538 (74%) individuals in the cohort study responded to the online survey, with 19 of respondents missing baseline data (e.g. age, gender, and occupation). Compared to non-respondents, survey respondents were significantly older, and more likely to identify as female sex and White race. Survey respondents also were significantly more likely to work in administrative roles and less likely to work as a physician or registered nurse. After removing participants who had missing data on burnout and key independent variables (e.g. age, gender, and PROMIS measures), a total of 2,411 participants were included in the current analyses (data flow diagram in Supplemental Figure 1). Table 1 contains demographic and work-related characteristics of the sample. The majority (*n* = 1,950, 80.88%) of HCWs in this study identified as White, Non-Hispanic race, and female sex (*n* = 1,986, 82.37%), and roughly one third (*n* = 747, 30.98%) were between ages 30–39 years old. Registered nurses (*n* = 650, 26.96%) accounted for the largest single occupation of HCWs, followed by physicians (*n* = 431, 17.88%) and administrators (*n* = 416, 17.25%). The mean score of burnout as measured by the OLBI was 36.87 (SD = 7.65), with 53.38% participants classified as having burnout based on cutoff scores. The highest proportion of HCWs with burnout were registered nurses (63.54%), followed by physicians (53.60%) and other healthcare occupations (53.28%). The proportion of HCWs experiencing burnout also varied by age, sex, and self-reported COVID-19 infection and exposure (Table 1). For instance, a significantly greater proportion of HCWs reported burnout identified as female sex (*n* = 1,098, 55.29%, *p* < 0.001) than those who identified as male sex (*n* = 189, 44.47%). Further, 71% (*n* = 251) of HCWs ages 18–29 years old were classified as having burnout, and the proportion of HCWs with burnout were significantly lower among 50–59 years-old (*n* = 203, 42.74%) and HCWs ages 60 and above (*n* = 73, 28.63%, *p* < 0.001). HCWs within single-person households also demonstrated the highest proportion of burnout (60.73%), with fewer individuals reporting burnout among two or more person households. Individuals with self-reported history of COVID-19 infection (*n* = 207, 58.81%) were significantly more likely to report burnout than those who did not report any history of COVID-19 infection (*n* = 1,080, 52.45%, *p* < 0.001).

**Table 1:** Demographic characteristics of HCWs and associations with burnout.

	**Burnout**			
Characteristics^*^	**Overall**	**Yes**	**No**	***P*-value**
**Age category, (** ***n*** **, %)**	2,411 (100%)	1,287 (53.38%)	1,124 (46.62%)	
				**<0.001**
18–29 y	353 (14.64)	251 (71.10)	102 (28.90)	
30–39 y	747 (30.98)	449 (60.11)	298 (38.89)	
40–49 y	581 (24.10)	311 (53.53)	270 (46.47)	
50–59 y	475 (19.70)	203 (42.74)	272 (57.26)	
60 + y	255 (10.58)	73 (28.63)	182 (71.37)	
**Sex, (** ***n*** **, %)**				**<0.001**
Female	1,986 (82.37)	1,098 (55.29)	888 (44.71)	
Male	425 (17.63)	189 (44.47)	236 (55.53)	
**Race/ethnicity, (** ***n*** **, %)**				0.57
Asian	205 (8.50)	111 (54.15)	94 (45.85)	
Hispanic/Latino	141 (5.85)	73 (51.77)	68 (48.23)	
Multiracial	39 (1.62)	24 (61.54)	15 (38.46)	
Non-Hispanic black	50 (2.07)	23 (46.00)	27 (54.00)	
Non-Hispanic white	1,950 (80.88)	1,039 (53.28)	911 (46.72)	
Other/NA	26 (1.08)	17 (65.38)	9 (34.62)	
**Household size (*****n*****, %)** ^*^				
1 person	303 (1.40)	184 (60.73)	119 (39.27)	**0.020**
2 persons	775 (34.36)	416 (53.68)	359 (46.32)	
3 persons	415 (18.35)	207 (49.88)	208 (50.12)	
4 persons	478 (21.13)	236 (49.37)	242 (50.63)	
≥5 persons	291 (12.86)	157 (53.95)	134 (46.05)	
**Occupation (** ***n*** **, %)**				**< 0.001**
Administrative Role	416 (17.25)	156 (37.50)	260 (62.50)	
Physician	431 (17.88)	231 (53.60)	200 (46.40)	
Registered Nurse	650 (26.96)	413 (63.54)	237 (36.46)	
Other	914 (37.91)	487 (53.28)	427 (46.72)	
**Patient contact (** ***n*** **, %)**				**<0.001**
Yes	2,001 (82.99)	1,120 (55.97)	881 (44.03)	
No	410 (17.01)	167 (40.73)	243 (59.27)	
**Vaccinated for COVID-19 (** ***n*** **, %)**				0.14
Yes	2,358 (97.80)	1,264 (53.60)	1,094 (46.40)	
No	53 (2.20)	23 (43.40)	30 (56.60)	
**COVID-19 patient exposure (** ***n*** **, %)**				**<0.001**
Yes	1,045 (43.34)	644 (61.63)	401 (38.37)	
No	1,366 (56.66)	643 (47.07)	723 (52.93)	
**Non-hospital COVID-19 exposure (** ***n*** **, %)**				**<0.001**
Yes	2,203 (91.37)	1,167 (52.97)	1,036 (47.03)	
No	208 (8.63)	120 (57.69)	88 (42.31)	
**Self-reported COVID-19 infection (** ***n*** **, %)**				**0.001**
Yes	352 (14.60)	207 (58.81)	145 (41.19)	
No	2,059 (85.40)	1,080 (52.45)	979 (47.55)	
PROMIS-depression, *m* (SD)	49.23 (7.25)	52.08 (6.53)	45.96 (6.64)	**<0.001**
PROMIS-anxiety, *m* (SD)	53.67 (7.86)	56.89 (6.72)	49.99 (7.44)	**<0.001**
PROMIS-positive affect, *m* (SD)	47.24 (8.64)	43.81 (7.36)	51.16 (8.31)	**<0.001**
PROMIS-meaning and purpose, *m* (SD)	53.52 (0.20)	49.71 (9.11)	57.89 (9.19)	**<0.001**

COVID-19 exposure captured as self-reported exposure to SARS-CoV-2 during healthcare work. COVID-19 vaccination was defined as self-reported first and second doses of the COVID-19 vaccine series. *P*-values are reported for chi-squared tests for group differences in demographic characteristics by burnout. Burnout was defined as having both OLBI-Exhaustion score ≥2.25 and OLBI-Disengagement score ≥2.1, based on Peterson, Demerouti (45). PROMIS measures are examined as *T*-scores (*M* = 50, SD = 10) of the general population.

* Values are *n* (%) unless stated otherwise.

The bold values indicate the number is statistically significant (*p* < 0.05).

A total of 359 respondents (14.89%) had a PROMIS-depression score more than one standard deviation above the mean, as well as 346 (14.35%) for the PROMIS-anxiety. Additionally, 14.35% (*n* = 346) of respondents scored one standard deviation below the mean for the PROMIS-positive affect, and 16.47% (*n* = 397) scored a standard deviation below the mean for the PROMIS-meaning and purpose score (see Supplemental Figure 2 for PROMIS measure distributions and cut-point descriptions).

In our multivariable linear regression model (Table 2) higher depression (coef = 0.15, SE= 0.03, *p* < 0.001) anxiety (coef = 0.25, SE = 0.02, *p* < 0.001) and lower positive affect (coef = −0.19, SE = 0.02, *p* < 0.001) and meaning and purpose (coef = −0.12, SE= 0.01, *p* < 0.001) scores were significantly associated with increased burnout scores. In addition, HCWs ages 60 years or older had lower burnout scores (coef = −3.25, SE = 0.46, *p* < 0.001) compared to individuals who were 18-29 years old; registered nurses (coef = 2.37, SE = 0.38, *p* < 0.001) and physicians (coef = 1.46, SE = 0.43, *p* = 0.001) reported greater burnout scores than individuals with administrative roles; and female respondents (coef = 1.07, SE = 0.25, *p* < 0.001) reported greater burnout scores than males. We also completed a logistic regression analysis which did not find any difference in factors associated with burnout as a dichotomous outcome (see Supplemental Table 1). Each unit increase in positive affect was associated with a 6% decrease in reported burnout (OR: 0.94, SE: 0.01, *p* < 0.001), and each increase in meaning and purpose was associated with a 5% decrease in burnout (OR: 0.95, SE: 0.01, *p* < 0.01).

**Table 2:** Multivariable linear regression analyses of the association between psychological measures and burnout (OLBI) adjusted for demographic characteristics, occupational group, and COVID infection history (*n* = 2,411).

**Variable**	**Coef**	**SE**	***t***	***P*-value**
Depression	0.15	0.03	5.86	**<0.001**
Anxiety	0.25	0.02	11.44	**<0.001**
Positive affect	−0.19	0.02	−10.48	**<0.001**
Meaning and purpose	−0.12	0.01	−8.33	**<0.001**
Patient contact (Y)	0.82	0.34	2.42	**0.016**
**Age category**				
18–29 years-old (ref)				
30–39 years-old	−1.01	0.35	−2.89	**0.004**
40–49 years-old	−1.53	0.37	−4.17	**<0.001**
50–59 years-old	−2.55	0.38	−6.59	**<0.001**
>60 years-old	−3.25	0.46	−7.11	**<0.001**
**Occupation group**				
Administrative Role (ref)				
Physicians	1.46	0.43	3.39	**0.001**
Registered Nurses	2.37	0.38	6.18	**<0.001**
Other	0.92	0.35	2.62	**0.009**
**COVID-19 exposure**				
No (ref)				
Yes	1.07	0.25	4.23	**<0.001**
**Sex**				
Male (ref)				
Female	1.11	0.31	3.64	**<0.001**

The bold values indicate the number is statistically significant (*p* < 0.05).

## Discussion

We found that among a cohort of HCWs who had been working since prior to the COVID-19 pandemic, higher scores on depression and anxiety were significantly associated with higher burnout. In contrast, positive affect and meaning and purpose were inversely associated with burnout scores. These statistically significant associations were stable even with the adjustment for other independent factors associated with burnout in our cohort (including age, female sex, occupational role, and patient contact, and COVID-19 exposure). We noted that burnout was prevalent (53.38%) in this cohort of HCWs at the time of data collection. Our findings also highlight the intersections of age, occupation, and sex in experienced depression, anxiety, positive affect, and meaning and purpose in association with burnout, with individuals with lower age, female sex, and occupational role as a physician or nurse with greater burnout.

Numerous studies have characterized the extent of burnout amid the COVID-19 pandemic and factors associated with higher rates of burnout. Our findings indicated that half of our participants were classified as having burnout as measured by the OLBI, (45) consistent with other estimates of burnout during the pandemic (54–57). For instance, Denning et al. (56) noted that 67% of HCWs in United Kingdom, Poland, and Singapore reported burnout based on the OLBI. Other studies reported even higher levels of burnout in HCWs, ranging from 75 to 79% (55, 57). These higher rates may be due to slightly different definitions of burnout or measurement at different points in the pandemic. Our findings are also consistent with other studies in that demographic and work characteristics associated with higher burnout scores included female sex (54), being a nurse, physician, or other patient-facing occupational role (56).

Our findings demonstrate that HCWs reported symptoms of depression and anxiety during the COVID-19 pandemic. This is not surprising as other studies have reported high rates of significant psychological distress by HCWs during the COVID-19 pandemic (11, 58–64). A meta-review of 40 systematic reviews of studies of HCWs during the pandemic found that depression and anxiety were the most prevalent mental health conditions (61). In concordance with our findings, another rapid review of the psychological impact to HCWs amid the COVID-19 pandemic noted that nurses who identified as female with contact to COVID-19 patients had the highest prevalence of mental health problems (65). Notably, given that surges in COVID-19 cases are mirrored with increased levels of burnout (66), the data collection period of our study may have led to lower reports of symptoms of depression, anxiety, and burnout in our sample, given the relative lull in COVID-19 cases occurring during that period.

We found a significant inverse association of positive affect and meaning and purpose with burnout in our cohort. Several studies, some completed before the pandemic, had similar findings. For example, one study of social workers in China in May 2021 found that positive affect was inversely associated with psychological distress, while negative affect was associated with higher with psychological distress (38). A study of primary care physicians in Spain conducted in 2013 found that positive affect mediated the relationship between lower resilience, lack of development, and burnout (67). Similarly, Barnett et al. observed that meaning and purpose was significantly inversely associated with burnout prior to the COVID-19 pandemic (37). One study also conducted prior to COVID-19 examining psychological well-being among HCWs noted that nurses, who were overwhelmingly female, reported significantly lower positive affect than other healthcare professionals (68). Similar studies have not been conducted during the COVID-19 pandemic. To our knowledge, our study is the first to report the associations of positive affect and meaning and purpose with burnout scores among a large cohort of multidisciplinary HCWs during the COVID-19 pandemic. While interventions exist to increase positive affect (31, 32, 69–71), researchers have yet to assess the efficacy, feasibility, and acceptability of these targeted interventions among HCWs.

This study has several limitations. First, our study participants largely identified as White race and female sex, and worked as registered nurses, physicians, or administrators, which may limit the generalizability of our findings to HCWs who are non-White, male sex or gender-non-binary, or work in other healthcare roles including staff with patient contact (e.g. custodial staff, nursing assistants, security, etc.). Further, despite high burnout reported among individuals within single person households, we did not include household size in our regression models due to high missingness of data for this variable in our sample. More research is needed to focus on the specific experiences with burnout and psychological wellbeing for these HCWs. The initial recruitment strategies for this cohort study included a focus on COVID-19 testing at the time of data collection, which may have also biased this sample towards HCWs who require in-person work associated with their role. We also utilized online recruitment and survey methods for data collection, which may have limited our ability to reach some HCWs for recruitment and engagement in this study. Of note, our utilization of PROMIS measures has limited our ability to determine clinically significant differences in psychological health and burnout, as PROMIS measures are not diagnostic tools for psychological well-being (52). Further, our analysis was conducted utilizing cross-sectional data, which precludes any ability to determine causality and directionality in the associations between psychological health and burnout. Future studies are needed to examine causal mechanisms of burnout associated with clinically meaningful differences in psychological health, as well as examinations of effective interventions to reduce burnout among HCWs.

## Conclusions

In conclusion, the prevalence of burnout, depression, and anxiety were high among HCWs during the 2nd year of the COVID-19 pandemic. However, positive affect and meaning and purpose were associated with lower levels of burnout. As we have noted in our findings, individuals who are younger, identify as female, and work in patient-facing occupational roles (e.g. RNs, MDs) were associated with higher rates of increased burnout in this study and would benefit from targeted interventions to promote resilience in these populations. Future research is needed to examine the association of specific domains of burnout- including exhaustion and disengagement- with depression, anxiety, meaning and purpose, and positive affect. Individual-level and systems-level interventional studies are needed to develop evidence-based interventions, potentially through increasing positive affect and sense of purpose, to reduce burnout and increase the resiliency and psychological wellbeing of the HCWs who are critical to providing health care before, during, and after current and future pandemics.

## Data availability statement

Data are available on reasonable request from the principle investigator CE, charlesnika-evans@northwestern.edu and if in accordance with institutional guidelines.

## Ethics statement

The studies involving human participants were reviewed and approved by Northwestern University Institutional Review Board (IRB). The patients/participants provided their written informed consent to participate in this study.

## Author contributions

JB, LH, CE, and JM contributed to conception and design of the study. JB organized the database and performed the statistical analysis with mentorship from TV. JB wrote the first draft of the manuscript. JB, MF, CL, and JM wrote sections of the manuscript. All authors contributed to manuscript revision, read, and approved the submitted version.

## Funding

This work was supported by the Northwestern University Clinical and Translational Sciences Institute (UL1TR001422), the Northwestern Memorial Foundation and a grant from the Peter G. Peterson Foundation Grant.

## Conflict of interest

The authors declare that the research was conducted in the absence of any commercial or financial relationships that could be construed as a potential conflict of interest.

## Publisher's note

All claims expressed in this article are solely those of the authors and do not necessarily represent those of their affiliated organizations, or those of the publisher, the editors and the reviewers. Any product that may be evaluated in this article, or claim that may be made by its manufacturer, is not guaranteed or endorsed by the publisher.
