# Supplementary material for: Psychological wellbeing and the association with burnout in a cohort of healthcare workers during the COVID-19 pandemic
Source: Front Health Serv. 2022 Oct 25;2:994474. doi: 10.3389/frhs.2022.994474 (PMC10012723; doi:10.3389/frhs.2022.994474)
Supplement: Supplementary file 1 [file Table_1.DOCX]

Supplemental Table 1: Logistic regression analyses of the association between psychological measures and burnout (OLBI-binned) adjusted for demographic characteristics, occupational group, and COVID infection history (n=2,411)

| **Variable** | **OR** | **SE** | **z** | **P-value** |
| --- | --- | --- | --- | --- |
| *Depression* | 1.02 | 0.01 | 1.69 | 0.091 |
| *Anxiety* | 1.08 | 0.01 | 7.09 | **<0.001** |
| *Positive Affect* | 0.94 | 0.01 | -6.86 | **<0.001** |
| *Meaning and Purpose* | 0.95 | 0.01 | -7.46 | **<0.001** |
| *Patient contact (Y)* | 1.34 | 0.21 | 1.88 | 0.060 |
| *Age category* |  |  |  |  |
| 18-29 years-old (ref) |  |  |  |  |
| 30-39 years-old | 0.70 | 0.12 | -2.17 | **0.030** |
| 40-49 years-old | 0.59 | 0.10 | -3.07 | **0.002** |
| 50-59 years-old | 0.43 | 0.08 | -4.68 | **<0.001** |
| >60 years-old | 0.27 | 0.06 | -6.10 | **<0.001** |
| *Occupation group* |  |  |  |  |
| Administrative Role (ref) |  |  |  |  |
| Physicians | 2.19 | 0.43 | 3.97 | **<0.001** |
| Registered Nurses | 2.36 | 0.42 | 4.91 | **<0.001** |
| Other | 1.71 | 0.27 | 3.36 | **0.001** |
| *COVID-19 exposure* |  |  |  |  |
| No (ref) |  |  |  |  |
| Yes | 1.27 | 0.15 | 2.12 | **0.034** |
| *Sex* |  |  |  |  |
| Male (ref) |  |  |  |  |
| Female | 1.42 | 0.20 | 2.46 | **0.014** |

Burnout was defined as having both OLBI-Exhaustion score ≥ 2.25 and OLBI-Disengagement score ≥ 2.1, based on Peterson, Demerouti (41).
