# Supplementary material for: Psychological wellbeing and the association with burnout in a cohort of healthcare workers during the COVID-19 pandemic
Source: Front Health Serv. 2022 Oct 25;2:994474. doi: 10.3389/frhs.2022.994474 (PMC10012723; doi:10.3389/frhs.2022.994474)
Supplement: Supplementary file 2 [file Table_2.DOCX]

Supplemental Figure 1: Data flow diagram for data cleaning

NM HCW Serology Parent Cohort N= 6,510

N= 3,538

Did not respond to burnout survey:

n= 919

NM HCW Extension Cohort

N= 3,538

Missing response to baseline data:

n= 19

Removed if missing the following:

OLBI total score: n= 98

PROMIS Bank v1.0 Meaning and Purpose t-score: n=58

Patient contact: n= 17

Age: n= 12

Dropped from sample:

Gender if selected “prefer to self-identify” n= 4

Total removed: n= 208

Responded to burnout survey:

n= 2,619

**Final included: n= 2,411**

Note: missing baseline data include demographic characteristics (age, sex/gender, occupation) collected upon initial enrollment within the Northwestern Medicine Healthcare Worker SARS-CoV-2 Serology Study.
