# Supplementary material for: Psychological wellbeing and the association with burnout in a cohort of healthcare workers during the COVID-19 pandemic
Source: Front Health Serv. 2022 Oct 25;2:994474. doi: 10.3389/frhs.2022.994474 (PMC10012723; doi:10.3389/frhs.2022.994474)
Supplement: Supplementary file 3 [file Table_3.DOCX]

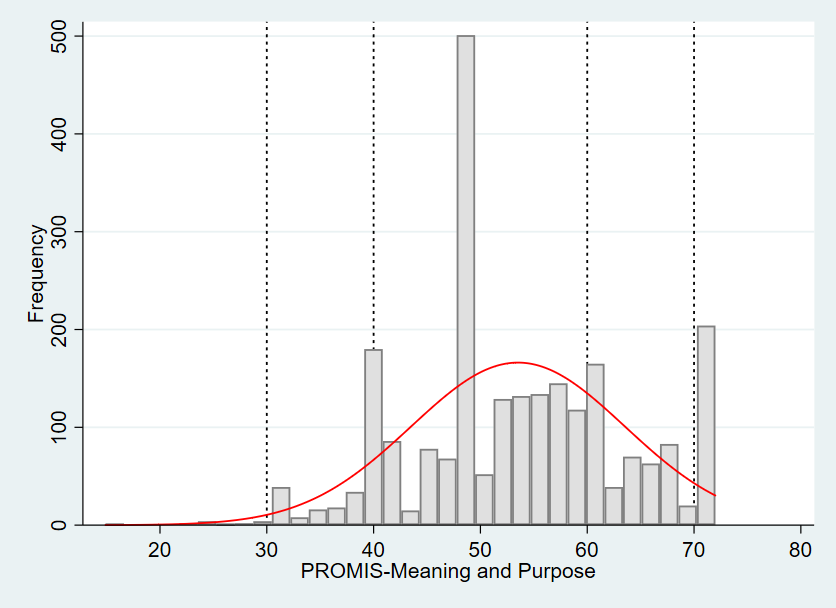

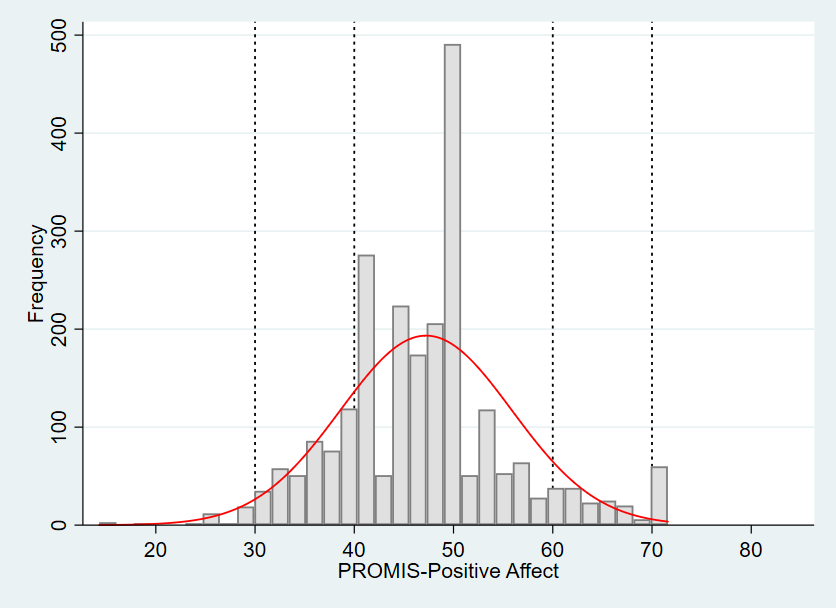

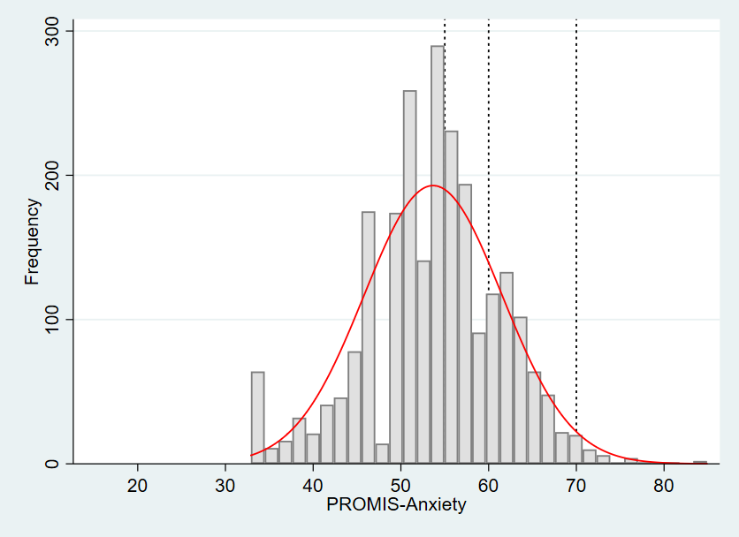
Supplemental Figure 2: Distribution of PROMIS measures with cut-points


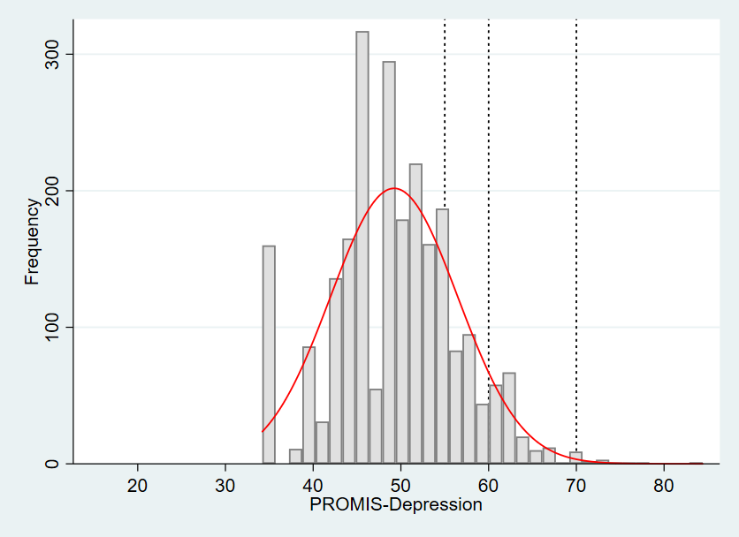


| **PROMIS T-Score Cut-Points Interpretation^47^** | | | | | |
| --- | --- | --- | --- | --- | --- |
| PROMIS-Depression and PROMIS-Anxiety | Normal limits  <55 | Mild  55-60 | Moderate  60-70 | Severe  >70 |  |
| PROMIS-Positive Affect and PROMIS-Meaning and Purpose | Very low  <30 | Low  30-40 | Average  40-60 | High  60-70 | Very High  >70 |
