# Supplementary material for: Psychological wellbeing and the association with burnout in a cohort of healthcare workers during the COVID-19 pandemic
Source: Front Health Serv. 2022 Oct 25;2:994474. doi: 10.3389/frhs.2022.994474 (PMC10012723; doi:10.3389/frhs.2022.994474)
Supplement: Supplementary file 4 [file Table_4.DOCX]

| Reviewer Comments | Response |
| --- | --- |
| 1. Contribution to the field: I suggest reference to specific examples of interventions in the workplace that have been shown to increase positive affect and meaning and purpose. | Thank you for this comment. We have added a statement in the introduction section to discuss previous interventions for HCWs to increase positive emotions.  The introduction now reads as (page 4, line 60-61):  *Additionally, there are few evidence-based interventions to reduce burnout, anxiety, and depression among HCWs. Of available evidence-based interventions for HCWs, these interventions largely focus on mindfulness or meditation-based interventions rather than broader psychological domains of well-being*.(25-28), as part… |
| 1. Background: narrative is very well structured, providing a clear overview of the key concepts and outlining clear hypotheses. | We are grateful for the compliment on our review of the relevant literature. |
| 1. Methods: were some groups of HCW more or less likely to attend testing? How representative is the sample? I see that this is addressed in the results and limitations section, but I also advise inclusion of some detail in the methods. | We have included a description of the representativeness of the original study cohort (Wilkins, 2021) as well as clarified the differences between the original cohort and the group included in this report.    The methods section now includes (page 4, lines 85-90):  *From May 28 to June 30, 2020, 38,127 Northwestern Medicine HCWs were invited in to participate in an employer-sponsored benefit that provided free SARS-CoV-2 serological testing, for which all HCWs were eligible. A total of 18,985 (49.8%) participated in the employer-sponsored serological benefit. HCWs enrolled in the benefit included 79.6% women and 74.9% non-Hispanic White, 9.7% Asian, 7.3% Hispanic, and 3.1% non-Hispanic Black workers, with a mean age was of 40.6 (SD:12.0) years old, and nurses represented the largest occupational group (n = 1794).*  The results section now reads as (page 7, lines 146-147):  Compared to non-respondents, *survey* respondents were significantly older, and more likely to identify as female sex and White race. Survey respondents also were significantly more likely to work in administrative roles and less likely to work as a physician or registered nurse. |
| 1. More a point of note than a revision request: It would be interesting to gain an insight into the two sperate elements of burnout (exhaustion and disengagement), as interventions may be tailored to address these different issues. Maybe something to note for future research. | We have added a statement to this point in our conclusions section for recommendations for future research to understand if different interventions are needed to target exhaustion versus disengagement as elements of burnout among HCWs.    Lines 250-251 on page 12 now read as:  *Future research is needed to examine the association of specific domains of burnout- including exhaustion and disengagement- with depression, anxiety, meaning and purpose, and positive affect*. Individual-level and systems-level interventional studies are needed to develop *evidence-based* interventions, potentially through increasing positive affect and sense of purpose, to reduce burnout and increase the resiliency and psychological well-being of the HCWs who are critical to providing health care before, during, and after current and future pandemics. |
| 1. Methods: More detailed information is required on the four psychological health measures. How was this information obtained, recorded, on what scale, using what instruments? | We have added a statement to provide information related to the PROMIS measures in the methods section.  Pages 6-7 lines 121-126 now includes the following:  *For the PROMIS depression and anxiety measures, the cut points for normal limits of anxiety or depression are T-score values below 55, mild from 55-60, moderate from 60-70, and severe above 70. For PROMIS positive affect and meaning and purpose, T-score value cut points for very high scores greater than 70, high from 60-70, average from 40-60, low from 30-50, and very low with values less than 30. See Supplemental Figure 2 for interpretation of these measures.*  We have also added details regarding data collection procedures to our methods section.  The methods section now reads as (page 5 lines 91-98):  Those that attended the serologic testing were recruited to enroll in the Northwestern Medicine Healthcare Worker SARS-CoV-2 Serology Study, a 1-year cohort study, which included *monthly, self-administered, online surveys* related to COVID-19 exposures, testing, symptoms, and vaccine intentions. In the original 1-year cohort study, 6,510 HCWs enrolled in the study. In June 2021, cohort participants were recruited to continue in an extension cohort study in which 3,538 consented to enroll in the ongoing extension cohort. This present analysis was conducted on psychological health and burnout data collected from the participants who enrolled in the extension and completed a *self-administered, online survey* between September 29-December 8, 2021. |
| 1. Statistical analysis: Why was burnout treated as a dichotomy for descriptive analysis and then as a continuous measure for regression models? It would be useful to understand the extent to which positive affect, purpose and meaning are associated with higher likelihood of crossing the threshold between non burnout and burnout, controlling for other factors. Suggest a second series of logistic models would enhance the paper. | We utilized a dichotomous measure for burnout based on previous literature for descriptive analyses and to guide selection of covariates for our linear regression model. We then conducted our linear regression analysis with burnout as a continuous measure such that we had sufficient power for our analyses. We also conducted logistic regression analyses that yielded similar findings to our linear regression analysis (see Supplemental Table 1). We have provided additional detail regarding these analyses in our results section and tables.  Our methods section now reads as (page 7, lines 130-136):  T-tests for comparison of mean values of continuous demographic and health characteristics and chi-squared tests for comparisons of proportions for categorical characteristics were used to assess burnout as a dichotomous variable for descriptive analysis *and to guide selection of covariates for our linear regression model.* For the main analysis, OLBI scores were analyzed as a continuous outcome measure in a multivariable linear regression model to assess the association between depression, anxiety, positive affect, and meaning and purpose on burnout with selected covariates *so that our analysis was sufficiently powered*.  Additionally, our results section now includes (page 9 lines 180-183):  *We also completed a logistic regression analysis which did not find any difference in factors associated with burnout as a dichotomous outcome (see Supplemental Table 1). Each unit increase in positive affect was associated with a 6% decrease in reported burnout (OR: 0.94, SE: 0.01, p<0.001), and each increase in meaning and purpose was associated with a 5% decrease in burnout (OR: 0.95, SE: 0.01, p<0.01).* |
| 1. Methods/ Results: What were the range of possible scores for burnout, anxiety, depression, positive affect, and meaning and purpose? This detail will have an important bearing on interpretation of results. | We provided additional detail regarding this point in our methods section and results tables.  The methods section (pages 6-7, lines 121-127) now includes the following:  *For the PROMIS depression and anxiety measures, the cut points for normal limits of anxiety or depression are T-score values below 55, mild from 55-60, moderate from 60-70, and severe above 70. For PROMIS positive affect and meaning and purpose, T-score value cut points for very high scores greater than 70, high from 60-70, average from 40-60, low from 30-50, and very low with values less than 30. See Supplemental Figure 2 for interpretation of these measures.* |
| 1. Related to previous points 6 and 7- the authors found higher burnout scores associated with positive affect and purpose and meaning, but are these changes clinically meaningful? Depending on the range of scores (point 7), coefficients may represent a small percentage change along the scale and may not be clinically meaningful if they occur at the lower end of the scale. This issue should be considered in the interpretation of results. | PROMIS measures were not developed as clinical tools for diagnostic purposes, and we do not consider burnout to be a clinical disorder. We have added a statement to discuss the limitations of our use of PROMIS measures in relation to clinical significance of our findings in the discussion section.  Our discussion section now includes the following (page 11, lines 233-238):  *Of note, our utilization of PROMIS measures has limited our ability to determine clinically significant differences in psychological health and burnout, as PROMIS measures are not diagnostic tools for psychological well-being.* Further, our analysis was conducted utilizing cross-sectional data, which precludes any ability to determine causality and directionality in the associations between psychological health and burnout. Future studies are needed to examine causal mechanisms of burnout associated with *clinically meaningful differences in* psychological health, as well as examinations of effective interventions to reduce burnout among HCWs. |
| 1. Thank you for the opportunity to review this paper on an important and timely topic which can make an important contribution to the field. This article is quite clearly written and its focus on prevalence of burnout, depression, and anxiety among Health Care Workers (HCWs) as well as positive psychological constructs is welcomed. This work can potentially make an important contribution to inform future interventions that seek to increase and foster positive affect and meaning among staff. | Thank you for this statement regarding our work. |
| 1. However, I have found the article is limited in the way the literature and data is used. The dearth and quality of direct literature in this area is of course a challenge and the data set is an acknowledged limitation but the authors could have considered the demographic characteristics of HCWs and associations with burnout much more systematically and linked back to the body of literature esp around the role of positive psychological constructs. Eg the older you get in this cohort the less likely there is burn out is an interesting finding that gets one sentence, single person households have the highest burn out but receives no comment, a gender analysis is missing which is strange given the focus of the study? | We include the results of the of the association of various demographic characteristics (e.g. age, sex) associated with burnout in our results section. We did not include number of people in a household due to high rates of missingness for that question. We have expanded the discussion of these results in the discussion sections.  Our results section now includes (page 8, lines 164-165):  *HCWs within single-person households also demonstrated the highest proportion of burnout (60.73%), with fewer individuals reporting burnout among two or more person households.*  Our discussion section includes (page 11, lines 231-232):  *Further, despite high burnout reported among individuals within single person households, we did not include household size in our regression models due to high missingness of data for this variable in our sample.*  Our discussion section also includes the following (page 10, lines 220-222):  *One study also conducted prior to COVID-19 examining psychological well-being among HCWs noted that nurses, who were overwhelmingly female, reported significantly lower positive affect than other healthcare professionals. (68)* |
| 1. The paper highlights that positive psychological constructs are related to resilience in the face of significant stress among HCWs - can you expand about the demographic and social characteristic of this cohort (ie those with positive psychological construct and low levels of burn out) and if possible the opposite of this cohort. The different aspects have been highlighted but not constructed in this version in a way that can inform interventions in a practical manner. | We have expanded on these points in our discussion to provide clarification. We note that selected demographic (e.g. age, sex) and occupational roles (e.g. RNs, MDs) were associated with higher rates of increased burnout in this study and would benefit from targeted interventions to promote resilience in these populations.  Our conclusions section now reads as (page 11, lines 247-249):  *As we have noted in our findings, individuals who are younger, identify as female, and work in patient-facing occupational roles (e.g. RNs, MDs) were associated with higher rates of increased burnout in this study and would benefit from targeted interventions to promote resilience in these populations.* |
